# User involvement in digital health: Working together to design smart home health technology

**DOI:** 10.1111/hex.12831

**Published:** 2018-10-05

**Authors:** Alison Burrows, Ben Meller, Ian Craddock, Fiona Hyland, Rachael Gooberman‐Hill

**Affiliations:** ^1^ Merchant Venturers School of Engineering University of Bristol Bristol UK; ^2^ Public Engagement, Research and Enterprise Development University of Bristol Bristol UK; ^3^ Bristol Medical School University of Bristol Bristol UK

**Keywords:** digital health, engagement, evaluation, impact, public involvement, technology development

## Abstract

**Background:**

Public involvement adds value to numerous aspects of health research, yet few studies have attempted to evaluate its impact on research. Evidence of such impact is needed to develop recommendations for best practice and ensure adequate resourcing.

**Aim:**

To evaluate public involvement within a large interdisciplinary Science, Technology, Engineering and Mathematics (STEM) research project that focused on digital health.

**Methods:**

The evaluation was conducted with members of the project's Public Advisory Groups (PAG) and with researchers who had participated in involvement activities. Two questionnaires were designed based on a public involvement value systems and clusters framework.

**Results:**

Responses from members of the PAG (n = 10) were mostly positive towards normative values, which include moral, ethical and political aspects of involvement in research, and towards values concerning the conduct of public involvement and best practices. Researchers’ responses (n = 16) indicated they felt that involvement was generally effective and increased the quality, relevance and generalizability of their work. However, their responses about the validity of involvement in research were varied. They also highlighted several challenges including how well public involvement impacted on research, how decisions made in the research might differ from the views generated from public involvement, and barriers to researchers’ participation.

**Discussion and conclusion:**

Our evaluation suggests that members of the public and the researchers value involvement. However, there is a need to consider how to embed public involvement to an even greater extent in STEM contexts and a need to address any barriers for researchers’ own involvement.

## 1 INTRODUCTION

The involvement of the public—which includes patients, carers, and health and social care service users^1^—in health research has gained prominence over the last decade. Much has been written about the benefits that public involvement can have at every stage of the research cycle,^2^, ^3^ including setting research priorities,^4^, ^5^ designing clinical trials^6^, ^7^ and placebos,^8^ and identifying treatment outcomes.^9^, ^10^ One widely accepted definition of this type of involvement, which is also adopted in this article, is research being carried out “with” or “by” members of the public rather than “to,” “about” or “for” them.^1^ Within this context, it is worth considering Arnstein's^11^ classic “ladder of citizen participation” model, which conceptualizes the degree of involvement from high to low. Although this model has since been refined to inform other ways to conceptualize public involvement in health research (eg,^12^, ^13^, ^14^, ^15^), the reality is research may include various forms of public involvement and these can change over time. It is therefore apt to distinguish three main levels of participation: consultation, where members of the public share their views and these views are used to inform decision‐making; collaboration, where an ongoing partnership is established between researchers and the members of the public so that decisions about the research are shared; and user controlled, where members of the public hold the power over all strategic decisions in the research.^16^

Recent developments in public involvement include guidance on how to achieve successful coworking,^17^, ^18^, ^19^ as well as recommendations on how to report activities.^20^ However, there is still a great need to build a research evidence base about the impact of involvement on research.^21^, ^22^ Doing so would contribute to ensuring the integrity of involvement activities, and enable the case to be made for support and adequate resourcing.^23^ Science, technology, engineering and maths (STEM) fields are an example of where involvement remains a “work in progress,” struggling to compete for time and resources.^24^ One explanation for why public involvement is less firmly established within STEM is a relatively recent and deliberate departure from a one‐way communication agenda, whereas in arts, humanities and social sciences, it is rooted in a tradition of participatory research approaches. This is especially problematic given that many STEM fields are heavily involved in the development of a range of digital heath solutions, which are frequently championed as a means of delivering care and empowering people to manage their health. Research has uncovered a variety of barriers and facilitators that service users experience during engagement with digital health engagement strategies, which include but are not limited to engagement and recruitment approaches.^25^ However, at an even earlier stage, public involvement may struggle in such contexts owing to the need to demonstrate its value and impact in STEM. Conducting empirical evaluation of involvement takes further time and resource, but provides necessary evidence so that involvement can be prioritized alongside and embedded within STEM research.

Meaningful evaluations should reflect public involvement as part of the research process and, as such, must revisit its values and purposes.^26^ In an effort to map out the values associated with public involvement in health research, Gradinger et al^27^ developed a framework comprising three overarching value systems. These relate to (a) normative perspectives, which concern moral, ethical and political aspects of public involvement; (b) substantive perspectives, which concern the consequences of involvement; and (c) process‐related perspectives, which concern the conduct and best practices of involvement. These value systems then contain five value clusters pertaining to each of them (see Table 1). This framework enables a structured approach to identifying what values different stakeholders attribute to public involvement, thus helping to manage potential conflict within a project and its wider organizational context. Although originally developed in the context of health and social care research, the framework has wider relevance. This framework was subsequently used in a modified Delphi study with stakeholders in public involvement in research, to explore areas of consensus and conflict around the proposed value systems.^23^ That Delphi study highlighted existing shortcomings in substantive and process aspects of public involvement, which further support the need for robust evaluations of involvement to develop best‐practice standards.

**Table 1:** Value systems and value clusters, adapted from Gradinger et al^27^

(i) Normative value system	Empowerment, Rights, Change/Action, Accountability/Transparency, Ethical values
(ii) Substantive value system	Effectiveness, Quality/Relevance, Validity/Reliability, Representativeness/Objectivity/Generalisability, Evidence base
(iii) Process value system	Partnership/Equality, Respect/Trust, Openness/Honesty, Independence, Clarity

With these issues in mind, we conducted an evaluation of public involvement embedded within a large interdisciplinary STEM project that aimed to design a fit‐for‐purpose system for monitoring health‐related behaviours in the home. The main foci of the strands of involvement work described below were to inform research and design, but also to explore issues pertaining to the role‐out of the system into real homes.^28^ To the best of our knowledge, this is the first evaluation of public involvement in a digital health project that uses the framework of Gradinger et al^27^ In addition to reporting the outcomes of the evaluation, this article discusses the process of balancing the needs and the expectations of service user groups in a digital health project that was driven by several factors, including expectations of the funder, research targets, and development of a working system. We begin by describing the methods used, in the spirit of the GRIPP2 checklist for reporting involvement in research.^20^

## 2 METHODS

This article describes an evaluation of public involvement, for which we used the framework of Gradinger et al^27^ to evaluate several strands of involvement that were embedded within a large interdisciplinary STEM research project. The work described here was conducted at the University of Bristol (UK) and the Engineering Faculty Research Ethics Committee stated that ethical approval was not required, because the work comprised evaluation rather than research.

### 2.1 Context

The SPHERE project aimed to design a smart home system comprising a range of nonmedical sensors. The sensors would collect information about various behaviours or activities in the home without requiring the occupants to engage much with the sensors, which would be “passive.” For instance, the sensors would collect information about use of the kitchen and movement around the home. The project was organized into six technical work packages, three of which corresponded to different types of information that the system would collect: environmental information including temperature, humidity and use of utilities; video‐based information including quality of movement and silhouettes (no raw video was captured); and activity and location information captured through a wrist‐worn device. The fourth work package aimed to optimize energy use and transfer; the fifth work package would combine streams of information and apply analytic methods to infer activities and behaviours; and the sixth would integrate the technology and place (“deploy” or “install”) it into people's homes. Each of these work packages was led by at least one senior academic and included several postdoctoral researchers and PhD students. The SPHERE public engagement and involvement team were separate, comprising one academic lead (FH), one public engagement associate (BM) and two community engagement officers from an external partner organization; working alongside this team was a more research‐oriented user‐centred design team (AB and RGH), with whom they worked closely. Their work traversed the technical work packages, seeking to involve and collaborate with researchers across the project. Mechanisms for public involvement comprised:

- Two Public Advisory Groups (PAG), which were set up at the start of the SPHERE project and met every 2 months to talk to researchers about their work and discuss topics including approaching potential participants, designing future studies, and features of the technology being developed. These meetings were organized and chaired by BM, who was the main point of contact for PAG members. One group comprised members of the general public and had 14 members; the other group comprised professionals with a background in social care and other professions that involve working with people in their homes (eg nurses, physiotherapists and housing officials) and had eight members. After each meeting, the groups’ feedback was circulated to all project researchers. The degree of involvement of the PAG corresponds to collaboration, as defined by INVOLVE.^16^
- A group called “Friends of SPHERE” made up of people who were interested in the project. This group signed up to receive newsletters and invitations to special events including demonstrations and discussion with each other and researchers. The aim of this group was to develop collaborative relationships between the research team and members of the public, and to establish partners in research and design activities. Five “Friends of SPHERE” events took place, which were attended by a total of 78 members of the public and 19 researchers (three of whom were work package leads). In terms of approaches to involvement defined by INVOLVE,^16^ this group corresponds to consultation with a view to developing collaboration.

The public engagement and involvement team also organized activities just with the researchers. These included annual workshops to discuss public involvement, and shorter lunchtime sessions held every 3 months to discuss issues emerging from public involvement activities.

### 2.2 Sample

The evaluation was conducted with two different groups: (a) members of the SPHERE PAG; (b) researchers who had participated in public engagement and involvement activities.

### 2.3 Questionnaire development

We chose to use a questionnaire approach to make it as easy as possible for members of the PAG and of the research team to participate. We were mindful in particular that members of the PAG were already generous in their time and that the researchers were already working at full capacity. We designed two questionnaires based on Gradinger et al's^27^ value systems and clusters framework for public involvement, one for completion by members of the PAG and a different one for completion by researchers. We explored the possibility of providing members of both groups the same questionnaire, but decided that it was more appropriate for the focus to be different. The questionnaire for PAG members (Appendix 1) therefore explored their experience of partnership and public involvement by focusing on the normative and process value systems; the questionnaire for researchers (Appendix 2) explored their experience of translation of public involvement activities into the research. The PAG questionnaire was structured in five sections: (a) consent for publication of anonymous quotations; (b) motivation and previous experience in public involvement; (c) normative value systems, with one question for each of the five value clusters; (d) process value systems, with one question for each of the five value clusters; and (e) additional comments. The questionnaire for researchers was structured in four sections: (a) consent for publication of anonymous quotations; (b) participant characteristics including the type of public engagement and involvement activities they participated in during their time on the SPHERE project, number of years they had worked in research and their previous experience of public involvement; (c) substantive value system, with one questions for each of the five value clusters; and (d) additional comments. The response options included five‐point Likert‐type scales and free‐text space to allow respondents to explain and give examples.

### 2.4 Data collection

This evaluation was conducted roughly at the midway point of the SPHERE project. The questionnaire designed for the PAG was posted to all 14 members at that time, and ten completed and returned them to the evaluation team. Of these, seven responded using the return envelopes provided and three returned their completed questionnaires at a group meeting.

The questionnaire designed for the researchers was completed by 16 people. At the time of the evaluation, approximately 30 academics and researchers were working in the project, including work package leads, postdoctoral and doctoral researchers. The questionnaire was first distributed during a lunchtime session attended by nine researchers all of whom returned a completed questionnaire. This questionnaire was also distributed via email and a further four researchers and three work package leads responded.

### 2.5 Collation and analysis of responses

Data were entered into Excel spreadsheets for collation and reviewed by the authors. Given the small sample size, responses to questions with Likert‐type response options were summarized in frequency tables and no further statistical analysis was performed. The qualitative material in the free‐text responses provided explanation and deeper understanding of experiences. These data were independently coded by AB, BM, FH and RGH, who subsequently discussed and refined them in a data analysis meeting. This process of critically revising the codes resulted in agreement of thematic categories, which were then applied to the data following a qualitative content analysis approach.^29^ A descriptive summary was developed based on these findings.

## 3 RESULTS

Results are reported separately for members of the PAG and the researchers. We present results by showing frequency of responses to the Likert‐type options, along with descriptions of responses to the free‐text options where appropriate.

### 3.1 The PAG's views

Ten members of the PAG returned completed questionnaires. Of these, five people reported no previous involvement in research; two people had been involved in clinical research; the remaining three people had previous experience of research or community engagement. Respondents were motivated to join the PAG because they felt their contribution could provide benefits to themselves (three people) and to others (six people), they supported the aims of the project (four people), and they felt they could provide specific insights that would lead to relevant and realistic outputs (four people).

#### 3.1.1 Normative value system

Table 2 summarizes how members of the PAG responded to Likert‐type questions about each of the value clusters within the normative value system. This table shows that members of the PAG scored their involvement in the project favourably for empowerment, rights and transparency. Specifically, responses indicate that members of the PAG felt moderately or highly empowered by their involvement; they felt that the public plays an important role in influencing this research; and there was consensus that the research team was transparent about their work.

**Table 2:** Frequency distribution of responses to Likert‐type questions about normative value system (N = 10)

Value	Question	Not at all	Slightly	Somewhat	Fairly	A great deal	No opinion	No response
Empowerment	3.1 To what extent do you feel able to make a contribution to the SPHERE project?	0	0	5	0	5	0	0
Rights	3.2 To what extent do you feel members of the public should be involved in shaping decisions in the SPHERE project?	0	0	2	0	7	0	1
Change/Action	3.3 To what extent do you feel the direction SPHERE has progressed in reflects your input?	0	1	4	1	4	0	0
Accountability/Transparency	3.4 To what extent do you feel able to ask any type of question to the SPHERE team?	0	0	0	0	10	0	0
Ethical values	3.5 To what extent do you feel your involvement in SPHERE has contributed to raising researchers’ awareness of ethical issues?	1	0	3	2	3	0	1

Responses to questions about Change/Action and Ethical values were slightly more diverse. The respondent who gave a lower score to Change/Action explained “I do not feel I have contributed a great deal but I have tried to ensure that a general approach is followed rather than concentrating on specific problems” (P5). Similarly, the members of the PAGs who gave a low score or no answer to the question about Ethical values suggested that this was due to their individual input in this area (“I don't feel that my knowledge in this field is enough to make any useful contribution”, P6). Overall, respondents felt personal perspectives, diverse experiences and early input were key to developing an appropriate system. Respondents also gave concrete examples of how their input had led to changes within the project (“Researchers gave demonstrations of their ‘inventions’ and listened to feedback. We were taken to the [demonstrator house], as requested. Researchers made feedback more friendly/less technical. Listened to ideas about watch/arm rest charger—things older people know,” P3) and raised awareness of ethical issues (“I believe we have been the ‘common sense’ element, giving examples and either questioning or reassuring. We have asked questions that make people/researchers think an issue through,” P3).

#### 3.1.2 Process value system

Table 3 summarizes how members of the PAG responded to Likert‐type questions about each of the value clusters within the process value system. Members of the PAG gave positive responses to questions about Partnership/Equality, Respect/Trust, Openness/Honesty, Independence and Clarity, with the majority giving the highest score.

**Table 3:** Frequency distribution of responses to Likert‐type questions about process value system (N = 10)

Value	Question	Not at all	Slightly	Somewhat	Fairly	A great deal	No opinion	No response
Partnership/Equality	4.1 To what extent do you feel the Advisory Group meetings reflect your interests about SPHERE?	0	0	3	0	7	0	0
Respect/Trust	4.2 To what extent do you feel listened to as a member of the SPHERE Advisory Group?	0	0	1	0	9	0	0
Openness/Honesty	4.3 To what extent do you feel the SPHERE Advisory Group is an open and fair forum for participation?	0	0	1	0	9	0	0
Independence	4.4 To what extent do you feel comfortable to express an opinion that is different to those of SPHERE researchers?	0	0	2	1	7	0	0
Clarity	4.5 To what extent do you feel you have a clear role within SPHERE?	0	0	1	0	8	0	1

Free‐text responses indicated that members of the PAG felt listened to (“Any comments were listened to, even if it turned out not to be something that was needed by the project,” P1) and this extended to beyond the meetings (“The opportunity to express thoughts and ideas by email, telephone or mail gave everyone the chance to be listened to,” P6). The PAG meetings were described as inclusive and open discussion forums (“BM ensures we all have time to speak and ensures clarity by questioning or restating. Also BM gets a response the next meeting if there is an unresolved issue or question,” P3). However, one respondent wrote “Researchers are experts in the field and I would be very reluctant to disagree with their opinions” (P6). Feedback included the need for more diversity among PAG members (“I think it would benefit from a more ethnically diverse composition and perhaps people who use the label ‘disabled’ to ensure a mix of opinions and cultures are represented,” P3).

### 3.2 The researchers’ views

Sixteen people completed the researchers’ questionnaire. Of these, thirteen were doctoral or postdoctoral researchers with between 3 and 18 years of research experience; the remaining three respondents were work package leads with between 22 and 28 years of research experience. Seven respondents reported they had little (one person) or no experience of public involvement in research before working on the SPHERE project; five respondents reported previous experience mostly through user testing and usability evaluation; a further three respondents described examples of sharing their research with the public rather than involvement, and one person gave no answer. Since joining the SPHERE project, all respondents had either directly taken part in public involvement activities (fifteen people) such as meeting with the PAG or other events where they demonstrated and discussed their work with the public, or taken part in activities such as workshops (fourteen people) where feedback from the public was discussed with a view to informing ongoing research and development. In the following sections, the term “public engagement” is sometimes used instead of public involvement.

#### 3.2.1 Substantive value system

Table 4 summarizes how SPHERE researchers responded to Likert‐type questions about each of the value clusters within the substantive value system. This table shows the researchers gave mostly positive responses to questions about Effectiveness and Quality/Relevance. Free‐text responses to the question about Effectiveness indicate that researchers found their experiences of public involvement in SPHERE surprising (“It's easy to try to imagine what public opinions will be, but on actually hearing them surprises are always thrown up. It's very easy to get caught up on something […] that turns out not be a problem and easy to miss things that turn out to be critical,” R5), as well as stimulating empathic thinking (“As a researcher, the public [engagement] activities have make me think a lot [about] the user's angle,” R1). Free‐text responses about Quality/Relevance show the researchers felt that public involvement had increased the value of the research by uncovering new research problems, refining existing ideas, and ultimately generating more appropriate outputs (“The public engagement activities help us build a deployable‐research mentality,” R3).

**Table 4:** Frequency distribution of responses to Likert‐type questions about substantive value system (N = 16)

Value	Question	Not at all	Slightly	Somewhat	Fairly	A great deal	No opinion	No response
Effectiveness	3.1 To what extent do you feel your interactions with the public through SPHERE Public Engagement^a^ activities have shaped your thinking about how health technologies will have to develop to be successful in people's homes?	0	0	5	6	4	0	1
Quality/Relevance	3.2 To what extent do you feel that SPHERE Public Engagement activities have contributed to more appropriate and relevant outputs?	0	0	4	10	2	0	0
Validity/Reliability	3.3 To what extent do you feel the public's views are a valid source for shaping the direction of SPHERE as a whole?	0	3	6	3	4	0	0
Representativeness/Objectivity/Generalizability	3.4 To what extent do you feel your Public Engagement experiences have contributed to learning that could be useful in future work?	1	1	5	5	4	0	0
Evidence base	3.5 If you could travel back in time to when you began working in SPHERE, to what extent would you make changes to the Public Engagement strategy?	3	3	5	0	0	3	2

a This term was used in the questionnaires instead of public involvement.

Responses about the Validity/Reliability of public involvement as an item from the framework of Gradindger et al^27^ were more diverse. Free‐text responses of the three respondents who gave lower scores to this question highlight the tension between the value of researchers’ knowledge and knowledge held by members of the public (“Scientific expertise should always be considered a more valid source for shaping research than public views,” R3). These respondents were researchers with at least 7 years of research experience, but who had no prior experience of public involvement in research. Answers also indicated that researchers felt a need to adhere to the existing plans for the research programme that had been defined and funded as such (“SPHERE was funded for having asked certain research questions so these should be answered objectively. However, the technology that SPHERE aims to create can only exist in a form of symbiosis with ‘the public’ who will ultimately be the benefactors of a successful project. Hence, their input is important even if over time it may change and become more aligned with SPHERE's needs,” R12). Other responses echo this sentiment that public involvement in research is “a two‐way street” (L3), where the public's views are “important, to some extent” (R6) and “should be used as a reality check” (R8).

Responses to the question related to Representativeness/Objectivity/Generalisability were mostly positive. The two respondents who gave lower scores on the Likert‐type options either did not give a free‐text response, or explained in their response that they felt their experiences of public involvement had not provided them with new insights beyond those already understood within the project. In the free‐text responses, researchers gave examples of knowledge they had gained through public involvement that would be transferable to other research contexts such as not using technical language or jargon when communicating with diverse audiences. The last question, which addressed issues around Evidence base, generated the highest number of abstentions due to no opinion or no response (five in total); these respondents explained that they were either satisfied with the delivery of public involvement in SPHERE or did not feel they knew enough to provide a useful answer. This question was phrased such that the lower end of the Likert‐type options (selected by six people in total) indicates researchers were mostly satisfied with the way in which the involvement took place within the project. The free‐text responses focused on areas of improvement to achieve more robust outputs from involvement activities, which included how well the public involvement work was able to influence the research (“The path between the public's viewpoints to the work packages has always been incongruous. […] As an example, I need to think for a minute or two to recall directives that were imposed on the research we perform that followed directly from public engagement, but I can easily recall a number of cases where work packages made decisions that felt—to me at least—contrary to the mood reported in the reports that we received from public engagements,” R13). Similar feedback was also reported in the additional comments section of this questionnaire, where one researcher wrote that they felt the public's views were not always taken on board because other team members “are not present at events or they base their opinion on conversations they've had instead of looking at the overall feedback” (R6).

Another issue raised in this section was that the project's involvement activities sometimes felt like a burden to researchers and could disturb work‐life balance, especially when these activities were carried out during evenings or weekends. One researcher wrote: “These events are on weekends/evenings and no time off in lieu is offered for participating. Many of us have private lives, families, children and working on weekends with no real benefits in return disturbs work‐life balance” (R6). Another researcher suggested “maybe rewarding also researchers for their participation” (R9), which could mean that they see public involvement as additional to their work load.

## 4 DISCUSSION

This work aimed to evaluate the impact of public involvement in a digital health project, as experienced by members of its PAG and by its researchers with STEM backgrounds. Through use of the value systems framework of Gradinger et al,^27^ the evaluation indicated that members of the PAG found public involvement in the project to be mostly positive in terms of normative and process values. Members of the PAG described several good practices that ensured they felt listened to within the project, such as seeing changes made as a direct result of their input, being able to express their thoughts outside of meetings using diverse means of communication, and having their questions answered appropriately. Within the PAG processes, the PAG convenor (BM) worked to develop rapport between all those involved, including researchers and members of the PAG. We suggest that this may have had a positive impact on public involvement contributors’ views about the activity, in keeping with other evaluations.^30^ The researchers generally found their experience of involvement to be useful and felt it had increased the quality, relevance and generalizability of their work. However, their responses also indicated a need to consider how best to enable the involvement to have impact more deeply, as there were some research decisions that did not always accord with the views from involvement activities. In some ways, this is not necessarily a problem, as members of the PAG were comfortable that the researchers possessed technical “expertise” and a key impact of the involvement was enhancement of empathy for the future “users” of the technology under development. While some researchers did express resistance to hand over or even share ownership of the research to public involvement contributors, it is also feasible that such views could be explained by the relative newness of the researchers to public involvement in research. This lends further evidence to calls to create an empirical evidence base of the impact of public involvement in research, which will pave the way to best‐practice standards.^21^, ^22^, ^23^

The trend towards more fluid collaborations between universities and external communities has uncovered challenges related to translating experiential learning and intellectual challenge into appropriate end‐of‐project outputs.^31^ Indeed, some researchers in our evaluation said that delivering research in line with the funded research agenda was the primary goal of their work and there was sometimes reluctance to alter plans on the basis of input from involvement activities. The focus was on developing a working system that could be replicated and rolled out into a large number of homes. Another study found that health researchers experienced similar tensions around the involvement of service users, strict deadlines, and the need to share power in research relationships.^32^ We suggest that the current research funding landscape could consider how best to allow for flexible research studies so that involvement can have meaningful impact on study development. Another area ripe for research is any interconnection between engagement with involvement and characteristics within the research community. Researchers in our evaluation indicated that it could be hard to accommodate activities that happened outside their usual working hours. This did not reflect an unwillingness to commit time to public involvement, but rather indicated that there might be real barriers to participation (eg caring commitments) that impact unequally on different members of the research community. An important area for future work could be to explore whether such barriers lead to unintended consequences or disadvantages for some, and to identify what steps could be taken to address these.

Some strengths and limitations need to be considered when interpreting our evaluation. The use of Gradinger et al's^27^ framework provided a research‐based structure and enabled us to focus on robust and meaningful values associated with public involvement. We thought carefully about whether a questionnaire approach was best, or whether we could use alternative approaches such as focus groups or one‐to‐one interviews. We chose a questionnaire based on a decision to make participation as easy as possible. It is of course possible that other approaches could have generated different or further views, but we were heartened by the depth of answers provided in the free text boxes. The provision of questionnaires was also advantageous, because people were able to complete the evaluation in a time and place of convenience to them. We chose to use a mixed methods approach, using a triangulation process^33^ that combined the collection of quantitative and qualitative information to obtain a more complete picture. This was for two reasons: first, we wanted to ensure the questionnaires were straightforward to complete, and we user‐tested them within the evaluation team; second, we thought it vital that we collected detail about the quantitative material. We found the quantitative material provided a snapshot of experiences and opinions, while the qualitative material provided depth and information that could enable improvement and change. This is in keeping with recommendations for the use of mixed methods approaches, and the complementarity of quantitative and qualitative information.^33^

The decision to develop different questionnaires for the researchers and for the PAG members was taken, as the substantive values associated with incorporating involvement into research were not obviously the domain of the PAG members. Although not all PAG members and researchers responded to our invitation to complete the evaluation, the diversity of backgrounds and the number who did provides confidence that the views captured reflect those of the wider group of PAG members and researchers. One caveat is that it is likely that the most engaged researchers were the ones who took part in the involvement activities. Additionally, we note that one of the evaluations was completed by a researcher who had not directly taken part in involvement activities. This might represent a failure on our part to make the events seem relevant and enticing to all researchers, or it might be that no amount of relevance or enticement would have encouraged some researchers to come. We did not formally collect information about why some researchers in the project were not involved in public involvement events, and this would be an excellent topic for further work. Informally, we understood that researchers who did not come to events felt that their time priorities lay elsewhere in their work. It is important to acknowledge that as an evaluation team we thought that public involvement is useful and important to the delivery of research that is grounded in the values and views of members of the public. We are aware that this might have affected our interpretation of the evaluation material, and this is why we used the framework of Gradinger et al^27^ as well as a robust approach to analysis.

In conclusion, public involvement in the project can be best described as “expedient,” as members of the PAG and researchers were involved in a process that was fit for purpose and deliverable. There is always scope to refine and improve involvement activities, and with more resource we would have conducted more coworking processes and explored how best to remove barriers to researchers’ involvement. The evaluation indicates that the members of the public who were involved felt that their views were valued and that they were listened to, and that researchers in a technology development environment valued involvement. However, the occasional instances of respondents who were less positive about their experience of public involvement within the project suggest there is still need for improved communication about the value of public involvement as well as for consideration of the drivers for research. We think that this evaluation and critical reflection on our work represent a large move forward in fostering and nurturing public involvement in a digital health project.

## CONFLICT OF INTERESTS

The authors know of no conflict of interests.
