# Supplementary material for: User involvement in digital health: Working together to design smart home health technology
Source: Health Expect. 2018 Oct 5;22(1):65–73. doi: 10.1111/hex.12831 (PMC6351410; doi:10.1111/hex.12831)
Supplement: Supplementary file 1 [file HEX-22-65-s001.docx]

Evaluation of SPHERE Advisory Groups

We would be very grateful if you would take the time to answer the following questions about the SPHERE Advisory Group.

Please return the completed questionnaire in the enclosed return envelope as soon as possible**.**

# Part 1: Publication of anonymous quotations from your responses

We would also like to ask your permission to publish quotations from some of the responses to the questions. These would be anonymous, in other words, your name would not be published next to the quotation.

| If you are **happy** for us to publish anonymous quotations from your answers please tick this box. |  |
| --- | --- |

| If you are **do not** want us to publish anonymous quotations from your answers please tick this box. |  |
| --- | --- |

# Part 2: About you

| 2.1 Thinking back to when you were invited to take part in the SPHERE Advisory Group, why were you interested in taking part? |
| --- |

| 2.2 Before taking part in the SPHERE Advisory Group, did you have any previous experience in public and patient involvement with research? If yes, please give details. |
| --- |

# Part 3: The role of the SPHERE Advisory Group

| 3.1 To what extent do you feel able to make a contribution to the SPHERE project?  **Not at all Somewhat A great deal No opinion**   \|  \|  \|  \|  \|  \|  \|  \| \| --- \| --- \| --- \| --- \| --- \| --- \| --- \|   Please can you explain your answer and give examples, if appropriate. |
| --- | --- | --- | --- | --- | --- | --- | --- |

| 3.2 To what extent do you feel members of the public should be involved in shaping decisions in the SPHERE project?  **Not at all Somewhat A great deal No opinion**   \|  \|  \|  \|  \|  \|  \|  \| \| --- \| --- \| --- \| --- \| --- \| --- \| --- \|   Please can you explain your answer and give examples, if appropriate. |
| --- | --- | --- | --- | --- | --- | --- | --- |

| 3.3 To what extent do you feel the direction SPHERE has progressed in reflects your input?  **Not at all Somewhat A great deal No opinion**   \|  \|  \|  \|  \|  \|  \|  \| \| --- \| --- \| --- \| --- \| --- \| --- \| --- \|   Please can you explain your answer and give examples, if appropriate. |
| --- | --- | --- | --- | --- | --- | --- | --- |

| 3.4 To what extent do you feel able to ask any type of question to the SPHERE team?  **Not at all Somewhat A great deal No opinion**   \|  \|  \|  \|  \|  \|  \|  \| \| --- \| --- \| --- \| --- \| --- \| --- \| --- \|   Please can you explain your answer and give examples, if appropriate. |
| --- | --- | --- | --- | --- | --- | --- | --- |

| 3.5 To what extent do you feel your involvement in SPHERE has contributed to raising researchers’ awareness of ethical issues?  **Not at all Somewhat A great deal No opinion**   \|  \|  \|  \|  \|  \|  \|  \| \| --- \| --- \| --- \| --- \| --- \| --- \| --- \|   Please can you explain your answer and give examples, if appropriate. |
| --- | --- | --- | --- | --- | --- | --- | --- |

# Part 4: How the SPHERE Advisory Groups work

| 4.1 To what extent do you feel the Advisory Group meetings reflect your interests about SPHERE?  **Not at all Somewhat A great deal No opinion**   \|  \|  \|  \|  \|  \|  \|  \| \| --- \| --- \| --- \| --- \| --- \| --- \| --- \|   Please can you explain your answer and give examples, if appropriate. |
| --- | --- | --- | --- | --- | --- | --- | --- |

| 4.2 To what extent do you feel listened to as a member of the SPHERE Advisory Group?  **Not at all Somewhat A great deal No opinion**   \|  \|  \|  \|  \|  \|  \|  \| \| --- \| --- \| --- \| --- \| --- \| --- \| --- \|   Please can you explain your answer and give examples, if appropriate. |
| --- | --- | --- | --- | --- | --- | --- | --- |

| 4.3 To what extent do you feel the SPHERE Advisory Group is an open and fair forum for participation?  **Not at all Somewhat A great deal No opinion**   \|  \|  \|  \|  \|  \|  \|  \| \| --- \| --- \| --- \| --- \| --- \| --- \| --- \|   Please can you explain your answer and give examples, if appropriate. |
| --- | --- | --- | --- | --- | --- | --- | --- |

| 4.4 To what extent do you feel comfortable to express an opinion that is different to those of SPHERE researchers?  **Not at all Somewhat A great deal No opinion**   \|  \|  \|  \|  \|  \|  \|  \| \| --- \| --- \| --- \| --- \| --- \| --- \| --- \|   Please can you explain your answer and give examples, if appropriate. |
| --- | --- | --- | --- | --- | --- | --- | --- |

| 4.5 To what extent do you feel you have a clear role within SPHERE?  **Not at all Somewhat A great deal No opinion**   \|  \|  \|  \|  \|  \|  \|  \| \| --- \| --- \| --- \| --- \| --- \| --- \| --- \|   Please can you explain your answer and give examples, if appropriate. |
| --- | --- | --- | --- | --- | --- | --- | --- |

# Part 5: Additional comments

| 5.1 Is there anything else you would like to say? |
| --- |

**Thank you!**
