# Supplementary material for: User involvement in digital health: Working together to design smart home health technology
Source: Health Expect. 2018 Oct 5;22(1):65–73. doi: 10.1111/hex.12831 (PMC6351410; doi:10.1111/hex.12831)
Supplement: Supplementary file 2 [file HEX-22-65-s002.docx]

Evaluation of SPHERE Public Engagement

To help make the SPHERE Public Engagement activities better in the future, we would be very grateful if you would take the time to answer the following questions. If you need extra space, please feel free to continue onto additional pages.

# Part 1: Publication of anonymous quotations from your responses

We would also like to ask your permission to publish quotations from some of the responses to the questions. These would be anonymous, in other words, your name would not be published next to the quotation.

| If you are **happy** for us to publish anonymous quotations from your answers please tick this box. |  |
| --- | --- |

| If you are **do not** want us to publish anonymous quotations from your answers please tick this box. |  |
| --- | --- |

# Part 2: About you

| 2.1 What is your gender?  [ ] Male  [ ] Female  [ ] Prefer not to say |
| --- |

| 2.2 Which of the following types of activities have you been involved in? Please tick all that apply.  [ ] A3 workshops  [ ] A3 lunchtime events (e.g. Cake Club, Pizza Club)  [ ] Public Advisory Groups  [ ] Festivals, events, and exhibitions  [ ] Public talks (e.g. patient and older people’s groups) |
| --- |

| 2.3 How many years have you been working as an active researcher (include time conducting a research degree, if applicable)? |
| --- |

| 2.4 What, if any, is your previous experience of public involvement in research? |
| --- |

# Part 3: Engaging with SPHERE Public Engagement activities

| 3.1 To what extent do you feel your interactions with the public through SPHERE Public Engagement activities has shaped your thinking about how health technologies will have to develop to be successful in people’s homes?  **Not at all Somewhat A great deal No opinion**   \| (1) \| (2) \| (3) \| (4) \| (5) \|  \| (0) \| \| --- \| --- \| --- \| --- \| --- \| --- \| --- \|   Please can you explain your answer and give examples, if appropriate. |
| --- | --- | --- | --- | --- | --- | --- | --- |

| 3.2 To what extent do you feel that SPHERE Public Engagement activities have contributed to more appropriate and relevant outputs?  **Not at all Somewhat A great deal No opinion**   \| (1) \| (2) \| (3) \| (4) \| (5) \|  \| (0) \| \| --- \| --- \| --- \| --- \| --- \| --- \| --- \|   Please can you explain your answer and give examples, if appropriate. |
| --- | --- | --- | --- | --- | --- | --- | --- |

| 3.3 To what extent do you feel the public’s views are a valid source for shaping the direction of SPHERE as a whole?  **Not at all Somewhat A great deal No opinion**   \| (1) \| (2) \| (3) \| (4) \| (5) \|  \| (0) \| \| --- \| --- \| --- \| --- \| --- \| --- \| --- \|   Please can you explain your answer and give examples, if appropriate. |
| --- | --- | --- | --- | --- | --- | --- | --- |

| 3.4 To what extent do you feel your Public Engagement experiences have contributed to learning that could be useful in future work?  **Not at all Somewhat A great deal No opinion**   \| (1) \| (2) \| (3) \| (4) \| (5) \|  \| (0) \| \| --- \| --- \| --- \| --- \| --- \| --- \| --- \|   Please can you explain your answer and give examples, if appropriate. |
| --- | --- | --- | --- | --- | --- | --- | --- |

| 3.5 If you could travel back in time to when you began working in SPHERE, what things would you suggest that the SPHERE public engagement team should do differently?  **Not at all Somewhat A great deal No opinion**   \| (1) \| (2) \| (3) \| (4) \| (5) \|  \| (0) \| \| --- \| --- \| --- \| --- \| --- \| --- \| --- \|   Please can you explain your answer and give examples, if appropriate. |
| --- | --- | --- | --- | --- | --- | --- | --- |

# Part 4: Additional comments

| 4.1 Are there other ways that you think the SPHERE public engagement team could find out about impact of the engagement on SPHERE? |
| --- |

| 4.2 Is there anything else you would like to say? |
| --- |

**Thank you!**
